# Is #cleaneating a healthy or harmful dietary strategy? Perceptions of clean eating and associations with disordered eating among young adults

**DOI:** 10.1186/s40337-019-0246-2

**Published:** 2019-06-03

**Authors:** Suman Ambwani, Meghan Shippe, Ziting Gao, S. Bryn Austin

**Affiliations:** 1 Department of Psychology, Dickinson College, P.O. Box 1773, Carlisle, PA 17013 USA; 2 Strategic Training Initiative for the Prevention of Eating Disorders based at Harvard T.H. Chan School of Public Health and Boston Children’s Hospital, Boston, MA USA; 3 Department of Social and Behavioral Sciences, Harvard T.H. Chan School of Public Health, Boston, MA USA; 4 Division of Adolescent and Young Adult Medicine, Boston Children’s Hospital, Boston, MA USA

**Keywords:** Dieting, Clean eating, Orthorexia nervosa, Eating disorder, Risk, Prevention

## Abstract

**Background:**

Although “clean eating” is widely propagated through social media and anecdotal reports in the popular press, there is almost no scientific research on this potentially risky dietary strategy. The current investigation explored definitions and perceptions of “clean eating” and its associations with indicators of disordered eating among diverse U.S.-based undergraduates.

**Method:**

Undergraduates (*N* = 148, *M*_age_ = 19.41 years, 70.3% women) were asked to define “clean eating” via an open-ended question and then read vignettes featuring five “clean” diets, all of which caused mild functional impairment across multiple domains. Participants rated the extent to which they believed the diet was 1) “healthy,” 2) reflective of “clean eating,” and 3) likely to be adopted by them. Finally, participants completed questionnaires to assess body appearance evaluation, obsessive-compulsive symptoms, eating disorder symptoms, and symptoms of orthorexia nervosa.

**Results:**

Open-ended responses indicated that participants defined “clean eating” in varied but overwhelmingly positive terms. Repeated measures ANOVAs indicated that the “meal substitution” vignette was perceived as the least healthy, least “clean,” and least likely to be adopted, whereas the “new” (balanced) diet vignette was rated the highest on these domains. Correlations among diet perceptions and indicators of disordered eating were positive and significant.

**Conclusions:**

“Clean eating” is likely a heterogeneous phenomenon that is viewed favorably by U.S.-based college students even when it is linked with functional impairment and emotional distress. Ongoing examination of “clean eating” could clarify the potential benefits and risks posed by this dietary strategy and thus inform eating disorder prevention efforts.

## Plain English summary

There is little scientific research on “clean eating” despite its frequent promotion in social and popular media. Because past research has shown that dieting increases risk for eating disorders (EDs), it is important to examine what “clean eating” means and understand how it is perceived by young adults. We asked 148 U.S-based undergraduates to define “clean eating,” and then showed them five fictitious stories about “clean diets,” each of which also caused problems in work, social, and emotional functioning. Participants rated these diets based on how “clean” and “healthy” they seemed to be, and their willingness to follow these diets. Finally, participants completed questionnaires measuring symptoms of EDs and other related phenomena. Results indicate that participants defined “clean eating” in varied but overwhelmingly positive terms. They viewed the “meal substitution” story as the least healthy, least “clean,” and least likely to be adopted, whereas the “new” (balanced) diet story was rated the highest on these variables. Importantly, participants’ perceptions of the stories were significantly associated with their own ED symptoms. Findings suggest that “clean eating” has many different meanings but is largely viewed favorably even when it causes problems in functioning, and positive impressions of “clean diets” may signal risk for disordered eating.

## Background

Although college students are at increased risk for eating disorders (EDs), exhibiting high rates of maladaptive attitudes and behaviors [1], subthreshold ED symptomatology [2], and positive screens for EDs [3], the factors that confer increased ED risk among this population are not well understood. One such factor may be the trendy “clean eating” diet, which although poorly defined in the scholarly literature, is widely disseminated through the popular media. A recent editorial drew attention to the pseudoscientific basis of “clean eating” and cautioned against the potential for harm, noting, “it is imperative for health providers to understand when an obsession with a healthy diet transforms from a solution to becoming part of the problem” (p. 668) [4]. Indeed, the paucity of scientific research on “clean eating” has contributed to an overall lack of clarity regarding the phenomenon: is it a healthful dietary strategy, or could it reflect increased eating disorder risk?

### Dieting, disordered eating, and orthorexia nervosa

Although dieting is a common behavior, particularly among adolescent [5, 6] and young adult women [7], it has previously been identified as a risk factor for the development of binge-eating symptomatology [8], particularly when combined with other risk factors such as family history, mood disorders, or substance use disorders [9]. In fact, research suggests that even following special diets (i.e., vegetarian, pescatarian, vegan/raw, paleo, or, gluten-free) may be linked with higher rates of EDs [10], and results from a recent systematic review suggest that following a therapeutic diet due to chronic illness (e.g., diabetes, celiac disease) also increases ED risk [11]. Indeed, one obstacle to understanding dieting behavior lies in the varied usage of the word “diet,” which can reflect a range of intermittent or chronic behaviors such as moderate/healthy behavior modification (e.g., increased consumption of nutritious foods), extreme dietary restriction (e.g., fasting), and everything in between (e.g., limiting carbohydrate intake) [12]. One such variant to dieting behavior, Orthorexia Nervosa (ON), although not currently listed as a diagnosis in the *Diagnostic and Statistical Manual of Mental Disorders* [13], reflects a clinically meaningful, pathological obsession with eating only healthy, “pure” foods [14] and likely represents a common [15] and potentially dangerous syndrome [16].

“Clean eating,” which emphasizes the consumption of healthy, “pure” foods, may also reflect susceptibility to a pathological fixation with healthy eating. Indeed, the National Eating Disorder Association’s informational webpage features a video about ON titled “The dangers of dieting and clean eating,” suggesting interchangeable language usage without clarity about the terms [17]. Similarly, one of the few empirical investigations of “clean eating” defined it as “eating behaviors that are centered on proper nutrition, restrictive eating patterns, and strict avoidance of foods considered to be unhealthy or impure”(p.1) [18], but then cited ON research, thereby conflating the two (arguably related) concepts. Recently proposed diagnostic criteria for ON highlight the propensity for dietary preoccupation to escalate and “involve progressively more frequent and/or severe ‘cleanses’ (partial fasts) [sic] regarded as purifying or detoxifying” (p. 16) [19] and others suggest that ON may represent a more extreme, more dysfunctional variant of “clean eating” [4]. Moreover, like ON, “clean eating” also appears to bestow an element of moral superiority [18]. For instance, as one physician noted, “the command to eat cleanly implies that everyone else is filthy, being careless with their bodies and lives. It comes with promises of energy boosts, glowing skin, spirituality, purity, and possibly immortality. But this nonsense is all based on a loose interpretation of facts and a desire to make the pursuit of wellbeing an obsessive, full time occupation” (p.1) [20]. Given the lack of evidence on “clean eating,” the potential for nutritional deficiencies conferred by this dietary strategy, and the dearth of scholarly literature regarding treatment options for ON [4], there is an urgent need to better understand the nature of this cultural phenomenon.

### What is “clean eating”?

The British Dietetic Association identified “clean eating” as their number 1 “worst celebrity diet[s] to avoid in 2017” [21], and their previous lists have included other so-called “clean” diets, such as Paleo, Sugar-free, and “Vegan before 6pm,” [22] and most recently, Raw Vegan diet, Alkaline diet, and a particular brand of nutritional meal replacement supplements [23]. “Clean eating” has largely been popularized via social media websites (such as Instagram), food blogs and books written by non-expert celebrities describing their personal diets and lifestyles [4, 24], and websites offering specific guidance for college students on “eating clean” [25–28]. Definitions of “clean eating” typically include elements such as eating local, “real” (non-processed), organic, plant-based, home-cooked foods [29], but often also tout more extreme strategies such as eliminating gluten, grains, or dairy [21]. Given the absence of a clear definition and strategies to assess “clean eating” [24], it is unclear why people pursue these types of diets. Indeed, there are likely heterogeneous motivations for pursuing “clean eating,” ranging from general health or wellbeing, to weight loss, to curing diseases [30], and proponents of “clean eating” often make far-fetched claims about its purported benefits. Popular magazines frequently share testimonials such as “…along with juicing six times per day, I was cancer-free in three months” [31], and note benefits ranging from radiant skin, weight loss, and reduced cholesterol, to eliminating diabetes, skin rashes, and migraines [32]. Although some do caution against taking “clean eating” to a point of fearing foods [29], wide-spread promotion and ambiguity regarding the nature of “clean eating” highlight the need for further investigation.

In terms of scientific literature, we were able to find only three empirical studies on “clean eating” to date.^1^ One experiment reported greater stigma directed toward a protagonist who followed a “clean living diet” than a control target [18], while a sociological visual content analysis of top posts deploying #cleaneating and #eatclean hashtags on Instagram noted that food was the focus in less than a quarter of the images, illustrating the breadth of the phenomenon, and characterized "clean eating" as an “embodied endeavor” because of its classification of the body as a symbol of health, morality, and social status [33]. Finally, a cross-sectional survey exploring attitudinal and behavioral differences among women who did and did not follow guidelines from “clean eating” websites reported more favorable attitudes toward “clean eating” and higher levels of dietary restraint (a risk factor for EDs) among those who adhered to dietary advice from the websites [24]. Taken together, the limited empirical literature on “clean eating” suggests that although this cultural phenomenon may be stigmatized in some capacity, it also likely signifies morality and status and is importantly linked with health-related attitudes and behaviors.

### “Clean eating” and the potential for harm

“Clean eating” is a potentially risky strategy in several ways. First, when taken to the extreme, “clean eating” could have negative health consequences that resemble those of an eating disorder, such as reproductive issues, amenorrhea, osteoporosis, bone fractures, irregular heartbeats, difficulties concentrating and depression [34]; indeed, the National Eating Disorders Association cautions that the health consequences of extreme fixation with ‘healthful’ eating resemble those resulting from anorexia nervosa [35]. Second, promoting extreme views such as “sugar is the enemy” [36] and the need to omit certain food groups without justification (i.e., in the absence of allergies or intolerances) may contribute to disordered eating attitudes and behaviors. Third, “clean eating” may mask already existing ED attitudes and behaviors and thereby limit access to care: for instance, one clinician cautioned, “at best, clean eating is nonsense dressed up as health advice. At worst, it is embraced by those with underlying psychological difficulties and used to justify an increasingly restrictive diet – with potentially life-threatening results” [34]. This observation is consistent with research reporting higher levels of dietary restraint among adherers than non-adherers to dietary advice from “clean eating” websites [24]. Finally, “clean eating” may contribute to misinformation amongst the general public: given the far-fetched health benefits claimed by its proponents, it could contribute to further confusion about nutrition and thus impact population health.

### Study aims

Given the heightened risk for EDs conferred by dieting and the proliferation of non-scholarly, popular media information about “clean eating,” there is an urgent need to investigate the nature of this cultural phenomenon among high-risk groups such as college students. Due to an absence of empirical research and clear definitions, the aims of this exploratory study were twofold: first, we sought to examine how the terminology of “clean eating” was defined and perceived, and second, we sought to understand how attitudes toward popular “clean diets” may be associated with eating disorder symptoms and related clinical phenomena.

## Method

### Participants

Participants (*N* = 148) were U.S.-based undergraduate cisgender women (*n* = 104), cisgender men (*n* = 42), a transgender man (*n* = 1), and one unreported gender individual (*n* = 1) at a U.S. small liberal arts college. They were 18 to 30 years old (*M* = 19.41, *SD =* 1.66) and self-identified their racial background as 60.1% White American or European American, 8.1% Black American or African American, 5.4% Asian American, .7% Native Hawaiian or Pacific Islander, 16.9% international, and 4.7% biracial or multiracial (.7% declined to answer and 3.4% indicated “other”). Regarding ethnicity, 6.8% of respondents self-identified as Hispanic/Latinx, 68.9% as non-Hispanic/Latinx, 20.3% indicated that the question did not apply to them, and 4% declined to answer. Participants self-reported height and weight to estimate sample Body Mass Index (BMI; kg/m^2^), which ranged from 16.6 to 44.9 (*M* = 23.4; *SD* = 4.7; IQR = 5.6) and were classified as follows: BMI < 18.5 = 5.4%, 18.5–24.9 = 60.8%; 25–29.9 = 18.2%; > 30 = 8.8%; missing = 6.8%. Participants were recruited through the Psychology Department Participant Pool and earned research credit for their participation. The research was approved by the Institutional Review Board (IRB) prior to data collection.

### Measures

#### Defining clean eating

Participants were asked the following open-ended question: *What does the term “clean eating” mean to you? In other words, how would you define “clean eating?”*

#### Multidimensional body-self relations questionnaire-appearance scales (MBSRQ-AS [37])

The 34-item MBSRQ-AS measures appearance evaluation, appearance orientation, body areas satisfaction, overweight preoccupation, and self-classified weight. Participants rate their level of agreement with statements such as “I constantly worry about being or becoming fat” and “I am on a weight-loss diet” on a 5-point Likert scale. They are also asked to rate satisfaction with different parts of their bodies (e.g., “Face (facial features, complexion),” “Mid torso (waist, stomach)”) on a 5-point Likert scale. In the present study, we focused on subscales assessing satisfaction with specific aspects of one’s physical appearance (Body Areas Satisfaction Scale; BASS; 9 items) and tendencies to focus on one’s weight, fat anxiety, and dieting (Overweight Preoccupation; OP; 4 items). Cronbach’s alphas for these subscales in the current study ranged from ɑ = .76 (OP) to ɑ = .83 (BASS).

#### Obsessive-compulsive inventory - revised (OCI-R [38])

The 18-item OCI-R is a short form of the OCI, designed to assess symptoms of obsessive-compulsive disorder (OCD). Participants rate their level of agreement with statements such as “I check things more often than necessary,” “I find it difficult to control my own thoughts,” and “I need things to be arranged in a particular way” on a 7-point Likert scale, with higher scores reflecting a greater degree of OCD symptoms. In the present study, Cronbach’s ɑ = .90.

#### Weight Bias internalization scale - modified (WBIS-M [39])

The 11-item modified version of the original Weight Bias Internalization Scale (WBIS [40]) measures the internalization of negative attitudes toward larger bodies toward one’s own self-evaluation without specifying the weight status of the respondent. Participants respond to questions such as “I am less attractive than most other people because of my weight” and “My weight is a major way that I judge my value as a person” on a 7-point Likert scale, with higher scores showing greater weight bias internalization. In the present study, Cronbach’s ɑ = .82.

#### Eating disorder examination - questionnaire short (EDE-QS [41])

The 12-item EDE-QS is a shortened form of the original EDE-Q [42] designed to quickly and reliably assess ED pathology. Using the prompt of “on how many of the past 7 days…” participants use a 0 to 3 scale to indicate frequency in response to items such as “Has thinking about food, eating or calories made it very difficult to concentrate on things you are interested in (such as working, following a conversation or reading)?” and “Have you had a sense of having lost control over your eating (at the time that you were eating)?” Higher scores reflect greater ED symptomatology. In the present study, Cronbach’s ɑ = .84.

#### Eating habits questionnaire (EHQ [43])

The 21-item EHQ assesses the extreme emphasis on healthy eating that is characteristic of ON, including, knowledge of healthy eating, problems associated with healthy eating, and feeling positively about healthy eating. Participants rate the accuracy of items such as “I only eat what my diet allows,” “I have made efforts to eat more healthily over time,” and “I go out less since I began eating healthily” on a 4-point Likert scale. Past research on undergraduate students suggests good convergent, discriminant, and criterion-related validity and reliability of the EHQ [43] with an overall Cronbach’s alpha of .90 [44]. Higher scores reflect greater ON symptomatology. In the present study, Cronbach’s ɑ = .88.

### Materials

#### Vignettes

Five experimental vignettes (see Appendix 1) were developed to capture heterogeneous descriptions of “clean diets” as described in the popular media [30], by the British Dietetic Association [21], and in published literature [20, 45]. Each vignette introduced the protagonist as participating in one of the following diets (accompanied by a brief definition of the diet): an alkaline diet, a vegan diet, a gluten-free diet, a meal substitution diet, and a “new” (balanced) diet. The development of the “new” (balanced) diet was based on the United States Department of Agriculture (USDA) 2015–2020 dietary guidelines, which recommend consumption of assorted vegetables, fruits, mostly whole grains, fat-free or low-fat dairy, mixed proteins, and oils, while limiting saturated fats, added sugar, and sodium [46]. The remaining elements of the vignettes drew upon past vignette-based research examining ON and “clean eating,” which previously mentioned college student status, negative impacts of eating behavior on social functioning, excessive time required by the diet, belief in the “purity” of the diet, experiencing negative emotions when the diet is disrupted, and interference with schoolwork [18, 47].

To maintain uniformity, other elements of the experimental vignettes were standardized, including the protagonist’s ambiguous gender. The vignettes intentionally avoided mention of weight or motivation underlying the diet (beyond the desire for health and purity) due to inconclusive evidence on the role of weight as a motivating factor for “clean eating” behavior. The protagonist did not have any intolerances or allergies that required a particular diet. Thus, the only difference across vignettes was in the name and definition of the diet followed. Given the prominence of social media in disseminating information about “clean” diets, each vignette was accompanied by a photograph from Instagram. Photographs were selected based on two standards: 1) accuracy: it matched the specific definition of the featured diet (and not other featured diets), and 2) social endorsement: it had been “liked” by over 500 people on Instagram.

#### Attention check

One multiple-choice question was posed after each vignette to ensure that participants had understood the definition of the diet that was provided. For instance, participants were asked to select one of the following options: “A meal substitution diet: a) Consists of skipping some meals and not others, b) Is the same thing as a juice cleanse, c) Consists of meal replacement bars/shakes/juices packed with proteins, vitamins, and nutrients, or, d) Is a combination of a juice cleanse and eating regular meals.”

#### Post-vignette questionnaire

For each vignette, participants were asked to use a 5-point Likert scale (*very slightly or not at all* to *extremely*) for a series of questions rating the extent to which they considered the featured diet to be “healthy” and indicative of “clean eating.” They were also asked to rate the likelihood that they would adopt the diet for themselves. Finally, participants rated a list of possible reasons why someone might engage in the featured diet, with the option to provide additional reasons for engaging in the featured diet.

### Procedure

Data were collected in small groups in a classroom setting, supervised by at least one trained research assistant. The study was entirely computerized and administered using Qualtrics software. Participants reviewed the consent form, and then proceeded with the demographic questionnaire, study instruction sheet, and were then presented with the open-ended question to assess their definition of “clean eating.” They were then presented with the five vignettes in randomized order, and each vignette was immediately followed by its corresponding attention check question and post-vignette questionnaire. Participants then completed the self-report questionnaires and received a debriefing information sheet at the conclusion of the study.

### Data analyses

Open-ended responses to the questions about defining “clean eating” were coded via “theoretical” thematic analysis [48]. A research assistant familiar with the popular media literature on “clean eating” (MS) reviewed the entire set of participant responses to gain familiarity with the data and then generated an initial list of codes for diet-related themes and broader impact related-themes. These initial themes were shared with the research team and revised to clarify the distinctiveness of the themes and minimize overlap across categories. The primary coder then reviewed participant responses for the presence or absence of the themes; responses were coded multiple times if they fit more than one thematic category. Review of the preliminary results by the research team highlighted a need to distinguish between dietary *additions* and dietary *restrictions* (rather than merely “dietary changes”) and to distinguish between *positive* and *negative* impacts (rather than merely “broader impacts”); the coding scheme was thus adjusted accordingly, a codebook (with detailed guidelines and examples) was created, and the data were re-coded by the primary coder (MS). The entire set of participant responses was then reviewed by a second member of the team (SA) to ensure that the coding system sufficiently captured the breadth of participant responses. After a consensus meeting, during which any areas of confusion in coding participant responses were clarified, it was determined that an additional category was required to capture participants’ frequent references to abstract, wellness-related benefits associated with “clean eating.” Thus, an additional category for “wellness” was added to the coding scheme and the data were re-coded by the primary coder (MS) one last time. A second coder (ZG) coded a randomly selected subset of the responses (*n* = 30, reflecting approximately 20% of the total responses) to determine interrater reliability.

Responses to the vignettes and their associations with other clinical phenomena were analyzed in SPSS Version 24. Given the hour-long expected duration for the study, those who finished the survey in less than 20 min were automatically excluded from data analyses (*N* = 5). Additionally, those who incorrectly responded to the attention check questions for the vignettes were excluded from subsequent analyses (*N* = 38). Successive repeated-measures ANOVAs compared reactions to the different vignettes among participants, and descriptive statistics summarized the perceived reasons for engaging in the diets described in the vignettes. Finally, correlation analyses assessed associations among perceptions of “clean eating” and indicators of clinical phenomena.

## Results

Results indicated a high degree of agreement across raters (Cohen’s kappa = .82, *p* < .001). Examination of participant responses to the open-ended questions indicated that most characterized “clean eating” as engaging in something positive to promote overall well-being (91%), for instance, noting that it “is more than eating for having an attractive physical shape. It has to do with taking care of oneself.” Under this broad category of “clean eating” as a contributor to overall wellness, sub-themes highlighted the addition of nutritious foods to one’s diet (46%; e.g. “[it] means eating non-processed and healthy foods. Eating organic fruits and vegetables as well as whole grains [that] are good for you. Sugars and fats are okay for you in moderation, especially if you are getting healthy sugars from fruits and healthy fats from nuts and other proteins”), and restricting consumption of a wide variety of foods (49%; e.g. “[it] refers to eating less processed foods...typically cutting out empty calories, processed sugars, saturated fats, etc.”). More specifically, in the context of *adding nutritious foods* to one’s diet, the most common responses highlighted plant-based (30%), organic/natural/non-GMO (18%), and protein (16%) as important components of “clean” diets. For instance, participants described “clean eating” as “eating healthy salads, and lean meats,” and “healthier foods, such as fruits, vegetables, whole grain products, and locally grown produce. ‘Clean eating’ can also refer to the consumption of organic produce, which is generally healthier for individuals.” In the context of *limiting consumption of unhealthy foods*, the most common responses highlighted processed/junk/fast food (37%), fats (16%), and sugar (16%) restrictions as components of “clean” diets. For instance, participants commented that “clean eating” involved “cutting out empty calories, processed sugars, saturated fats, etc.” and “choosing to eat foods that you see as more natural, instead of things that are heavily processed.” There was almost no reference to dietary “cleanses” in the context of “clean eating” (1%; e.g. “[it refers to] eating healthy, going through a cleanse”). In terms of perceived overall impact, participants largely characterized “clean eating” as having a good overall impact (59%), such as by noting, “I love clean eating because it makes me more energized and helps my body feel more alive and active.” A small subset of participants identified potentially harmful impacts of “clean eating” (5%) such as following a rigid schedule or ignoring bodily cues for food-related decisions; for instance, one noted: “to engage in ‘clean eating’ one must eat breakfast, lunch, and dinner at the appropriate times of the day. In other words, a person cannot skip any meals or overeat the number of provided daily meals just because they are hungry or because they are in the mood for a very large snack,” while another stated, “I would define clean eating as eating on a diet and strictly maintaining that diet.” (See Table 1).

**Table 1:** Examination of open-ended responses defining “clean eating”

Theme	Identification Rate
Strategy to promote well-being	91%
Involves dietary changes	68%
Involves dietary additions	46%
Fruits/vegetables/plant-based	30%
Attention to farming strategies (e.g., organic/natural/non-GMO)	18%
Attention to geography and/or environment (locally grown/sustainable/environmentally-friendly)	3%
Healthy/good fats	2%
Natural/ healthy sugars	1%
Whole grains/grains	9%
Proteins and meats (including lean proteins)	16%
Supplements or cleanses	1%
Involves dietary restrictions	49%
Processed/junk/fast food/preservatives	37%
Fats/grease/oils/deep-fried	16%
Sugar	16%
Gluten	1%
Calories	4%
Meats	2%
Nature of overall impact	61%
Positive broader impact	59%
Feeling of healthiness	1%
Good for you/well-balanced/nutritious/in moderation	57%
Attention to geography and/or environment (locally grown/sustainable/ environmentally-friendly)	3%
Improved weight/body shape/appearance	2%
Negative broader impact	5%
Rigid schedule/strict diet	6%
Ignores physical/bodily cues	1%
Other (e.g., unfamiliar with the term; reference to “clean plate club”)	9%

*Note.* Many participant responses spanned multiple thematic categories (62%)

**Fig. 1:**
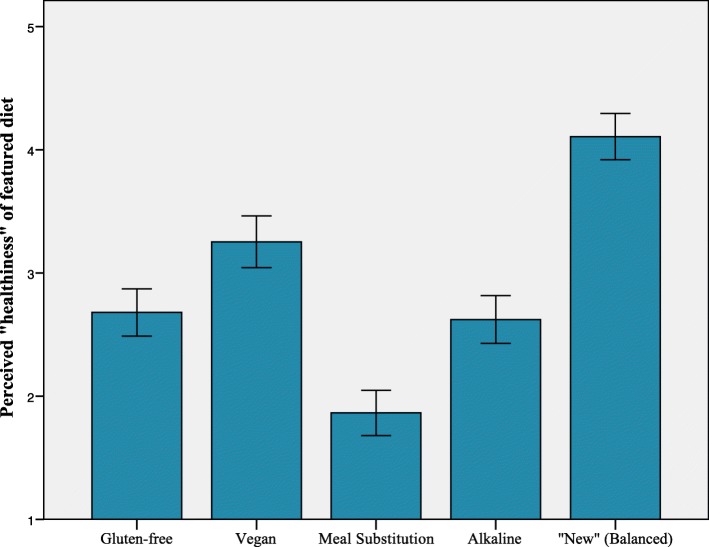
Mean ratings in response to the question, “To what extent would you consider this to be a “healthy” diet?” Error bars represent 95% CI

**Fig. 2:**
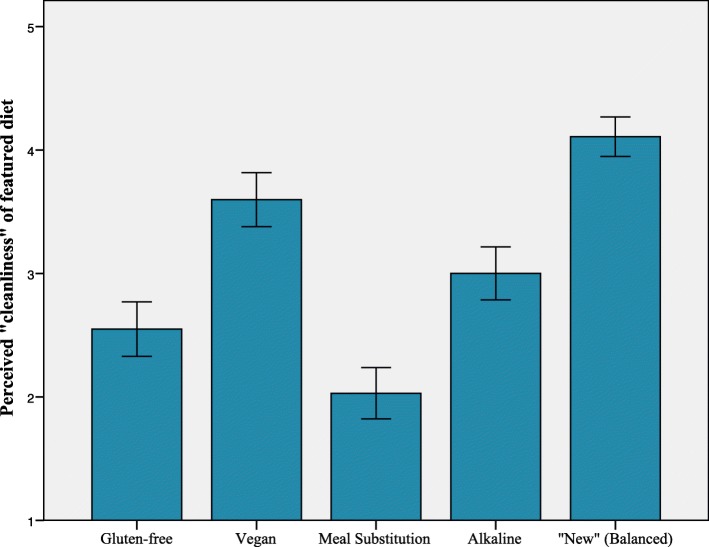
Mean ratings in response to the question, “To what extent would you consider this diet to be ‘clean eating’?” Error bars represent 95% CI

**Fig. 3:**
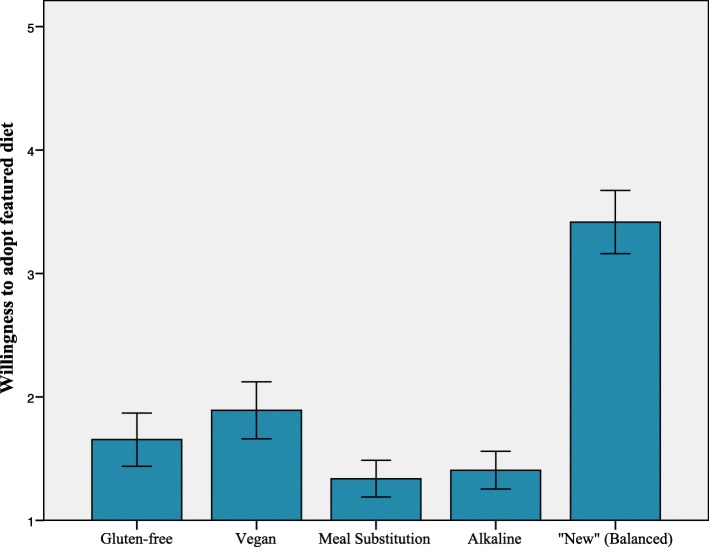
Mean ratings in response to the question, “To what extent would you seriously consider adopting this diet for yourself?” Error bars represent 95% CI

Successive repeated measures ANOVAs compared reactions to the gluten-free (GF), vegan (VG), alkaline-diet (ALK), meal substitution diet (MS), and “new” (balanced) diet (ND) among participants. Results indicate that *perceptions of healthiness* were significantly different across conditions *F* (4, 408) = 96.30, *p* < .001, ƞ_p_^2^ = .49, with ND rated as most healthy, VG rated as second most healthy, and MS rated as least healthy. Post hoc-tests using a Bonferroni correction indicated that GF and ALK did not significantly differ from each other; however, all other diet comparisons on perceived healthiness were significantly different (see Fig. 1). *Perceptions of cleanliness* were significantly different across conditions *F* (4, 404) = 88.95, *p* < .001, ƞ_p_^2^ = .47. Post hoc-tests using a Bonferroni correction indicated that all diets significantly differed from all other diets and were ranked in the following ascending order of perceived “cleanliness”: MS, GF, ALK, VG, ND (see Fig. 2). Finally, *willingness to adopt a particular diet* varied significantly across conditions; because Mauchly’s test was significant, we used the Greenhouse-Geisser correction, *F* (3.24, 324.28) = 77.66, *p* < .001, ƞ_p_^2^ = .44. Post hoc-tests using a Bonferroni correction indicated that ND was rated higher than all other diets (*p* < .001), and that VG was rated more favorably than MS and ALK; however, the other comparisons were not significantly different (see Fig. 3).

Descriptive statistics summarized the perceived reasons for engaging in the diets described in the vignettes (see Table 2). Overall, participants identified weight loss (*M* = 3.57, *SD* = .69), pursuit of health (*M* = 3.90, *SD* = .61), and to feel in control over one’s diet (*M* = 3.50, *SD* = .85) as the most important reasons for engaging in the featured “clean” diets, whereas social pressure (*M* = 2.51, *SD* = .99), feelings of superiority (*M* = 2.50, *SD* = 1.13), and improvement of skin (*M* = 2.87, *SD* = .82) were seen as less important reasons for engaging in these diets. Review of open-ended responses to the question about other reasons for engaging in these diets offered a range of possibilities, including the following: due to an eating disorder, to enhance athletic performance, due to taste preferences, due to social media/media impact, due to trendiness, due to animal welfare/ethics, due to fitness, due to religious or cultural practice, due to family tradition, due to environmental friendliness, due to mental health benefits, and to cleanse impurities.

**Table 2:** Perceived reasons for adopting various “clean” diets

	Type of Diet					
	Gluten-free 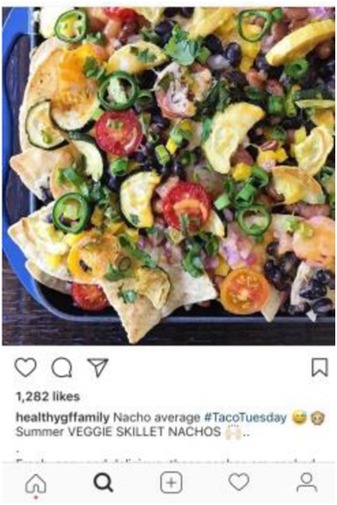	Vegan 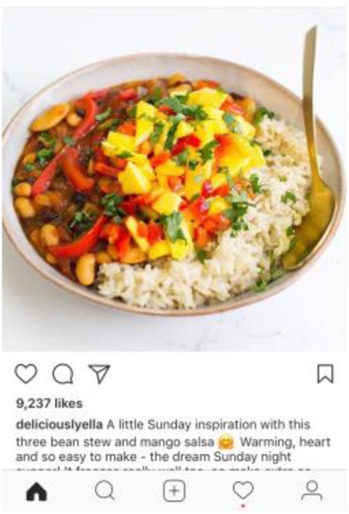	Meal substitution 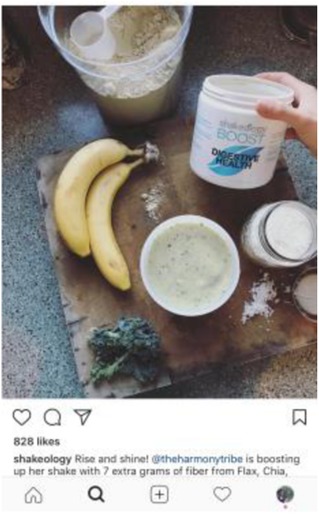	Alkaline 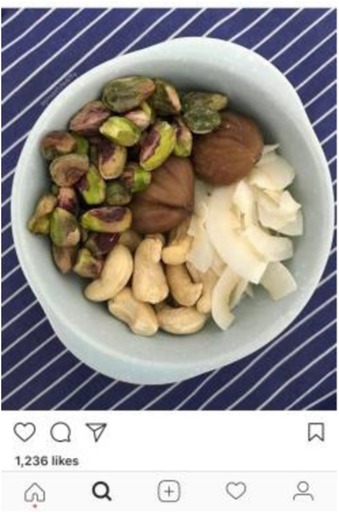	“New” (balanced) 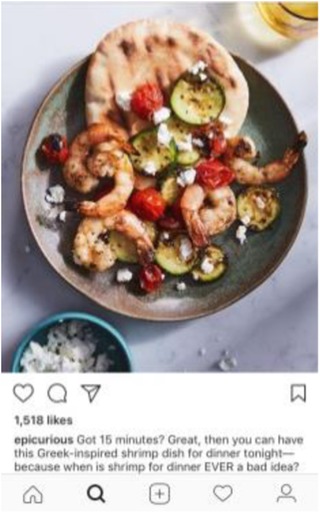	Across all 5 diets
**Description (excerpt): “Consists of…”**	…avoiding all foods that contain the protein gluten, which is found in grains such as wheat, barley, and rye.	…avoiding all meat, eggs, dairy, and any other animal products.	…a meal replacement (such as a protein/ vitamin/mineral-rich shake, bar, or juice) consumed in place of a normal meal.	…avoiding all acid-forming foods such as meat, fish, and most legumes; people who are on an alkaline diet consume food such as vegetables, fruits, and certain nuts and seeds to help balance the pH levels of the blood.	…eating assorted vegetables, fruits, whole grains, low-fat dairy products, and a variety of protein products (such as seafood, lean meats, eggs, and nuts), while limiting consumption of foods that are fried or have added sugars.	
**Reason for adopting diet**	M *(SD)*	M *(SD)*	M *(SD)*	M *(SD)*	M *(SD)*	M *(SD)*
For weight loss	3.10 *(1.22)*	3.40 *(1.17)*	4.20 *(0.91)*	3.15 *(1.10)*	3.97 *(1.01)*	3.57 *(0.69)*
To treat illness	3.28 *(1.23)*	3.18 *(1.20)*	2.23 *(1.20)*	3.21 *(1.20)*	3.19 *(1.22)*	3.03 *(0.85)*
To prevent illness	3.02 *(1.20)*	3.43 *(1.03)*	2.22 *(1.10)*	3.35 *(1.06)*	3.55 *(1.12)*	3.11 *(0.73)*
To be healthier	3.56 *(1.06)*	4.11 *(0.91)*	3.60 *(1.08)*	3.75 *(0.88)*	4.48 *(0.68)*	3.90 *(0.61)*
To feel superior to others	2.44 *(1.36)*	2.80 *(1.49)*	2.62 *(1.34)*	2.41 *(1.32)*	2.25 *(1.19)*	2.50 *(1.13)*
To improve skin	2.56 *(1.10)*	3.12 *(1.21)*	2.58 *(1.18)*	2.96 *(1.10)*	3.14 *(1.13)*	2.87 *(0.82)*
Due to social pressure	2.43 *(1.24)*	2.81 *(1.34)*	2.44 *(1.23)*	2.18 *(1.18)*	2.66 *(1.16)*	2.51 *(0.99)*
To feel in control of their diet	3.32 *(1.15)*	3.66 *(1.14)*	3.49 *(1.10)*	3.28 *(1.11)*	3.75 *(0.99)*	3.50 *(0.85)*

*Note.* For each of the dietary vignettes, participants were asked to answer the question, “if someone were to adopt this diet, to what extent might they do so for the following reasons” on a 1 (very slightly or not at all) to 5 (extremely) scale, using the options listed in the first column; participants also had the opportunity to provide alternative explanations through an open-ended “other” category

Finally, correlation analyses assessed linear associations among perceived healthiness of the five “clean” diets, perceived “cleanliness” of the five diets, and willingness to adopt the five diets, and clinical phenomena such as symptoms of ON, ED, OCD, weight bias internalization, and body image-related symptomatology. Results indicated positive associations among perceived healthiness, perceived cleanliness, and willingness to adopt with ON symptoms (EHQ; *r*s = .33–.61, *p*s < .01), with ED symptoms (EDE-QS; *r*s = .30–.41, *p*s < .01), and with overweight preoccupation (MBSRQ_AS_OP; *r*s = .26–.41, *p*s < .01). The positive association between perceived healthiness and weight bias internalization (WBIS_M; *r* = .19, *p* < .05) was also significant; other correlations were non-significant (see Table 3).

**Table 3:** Correlations among perceptions of “clean” diets and indicators of eating disorder relevant symptomatology

	Perceived cleanliness	Perceived healthiness	Willingness to adopt	EDE-QS	EHQ	OCI-R	WBIS-M	MBSRQ_ AS_BASS	MBSRQ_ AS_OP
Perceived cleanliness	–								
Perceived healthiness	.84***	–							
Willingness to adopt	.57***	.62***	–						
EDE-QS	.30**	.32***	.41***	–					
EHQ	.33***	.35***	.61***	.46***	–				
OCIR	.13	.12	.05	.20*	.07	–			
WBIS-M	.15	.19*	.16	.68***	.24*	.11	–		
MBSRQ-AS-BASS	.06	.02	.07	−.43***	.01	−.16	−.69***	–	
MBSRQ-AS-OP	.26**	.28**	.41***	.80***	.46***	.09	.56***	−.33***	–

*Note*. *p < .05; **p < .01; ***p < .001. Scores for perceived cleanliness, perceived healthiness, and willingness to adopt the presented diets were averaged across all five “clean” diet vignettes. EDE-QS is the Eating Disorder Examination Questionnaire Short; EHQ is the Eating Habits Questionnaire; OCI-R is the Obsessive-Compulsive Inventory-Revised; WBIS-M is the Weight Bias Internalization Scale – Modified; MBSRQ-AS-BASS and MBSRQ-AS-OP are the Body Areas Satisfaction and Overweight Preoccupation subscales from the Multidimensional Body-Self Relations Questionnaire Appearance Scales, respectively. Participants who completed the survey in less than 20 min (*n* = 5) or incorrectly responded to the attention check questions for the vignettes (*n* = 38) were excluded from the correlation analyses

## Discussion and conclusions

Although “clean eating” is widely promoted through social networks and popular media, there is a dearth of scientific research examining the nature of and attitudes toward this phenomenon. Given the increased risk for EDs conferred by dieting, there is an urgent need to better understand how ostensibly healthy diets may devolve into disordered eating. Our findings suggest that undergraduate students define “clean eating” in varied but overwhelmingly positive terms, with definitions that frequently highlight the perceived healthiness of this dietary strategy. Although past survey research suggests that somewhat fewer respondents (between 39.7 and 63.9%) exhibit positive views toward “clean eating” [24], differences in results may be attributable to the research question posed to participants: whereas we asked about *definitions of* “clean eating,” Allen and colleagues [24] inquired about *opinions toward* “clean eating.” Moreover, whereas we recruited U.S.-based undergraduates of all genders, Allen and colleagues [24] recruited young adult through older adult (ages 17–55 years) women; thus, observed differences may be attributable to sample variation in experiences and attitudes toward “clean eating” and reflect the high-risk nature of our sample.

Most of our participants were familiar with the term, “clean eating,” which reflects the high level of exposure to messaging about this dietary strategy. In terms of dietary composition, “clean eating” was perceived as heterogeneous in content, involving greater consumption of plant-based foods, organic, natural, non-GMO, unprocessed foods, locally grown, sustainable, environmentally-friendly foods, “good fats,” natural sugars, whole grains, and lean proteins, and reduced consumption (or elimination) of junk foods, fast foods, preservatives, fats, sugar, gluten, calories, and/or meats. These responses highlight a lack of shared understanding regarding the dietary content of “clean eating” and suggest that the term remains open to interpretation by consumers. It is noteworthy that definitions of “clean eating” largely did not refer to dietary supplements or cleanses, and that the “meal substitution” vignette (which most closely resembled a cleanse diet by its shared focus on consumption of nutrients through beverages) was perceived as the least healthy, least “clean,” and least likely to be adopted by participants. This finding was unexpected, given the lexical overlap between “clean” and “cleanse” and the frequent conflation of the two terms in popular media. One possibility for this distinction is that participants frequently identified “non-processed” and “natural” as important components of “clean eating,” and meal substitution diets frequently require consumption of processed products. Another possibility is there may be differences in the perceived duration of these dietary strategies: whereas “cleanses” are commonly marketed as short-term efforts, “clean eating” is often promoted as a longer-term lifestyle change. It may also be the case that cleanses are adopted as “corrective” behaviors (i.e., to counter the perceived ill effects of a poor diet, or ‘unclean’ eating), whereas “clean eating” is pursued as a “proactive” measure (i.e., to prevent the need for future ‘cleanses’ or other corrective behaviors). Future research that more explicitly compares reactions to “cleanse diets” versus “clean eating” could clarify similarities and differences between these dietary strategies and thereby inform health-promotion interventions.

Present findings indicate a potential for harm associated with favorable attitudes toward an extreme “clean eating” regimen. First, although our “new” (balanced) diet vignette did feature a diet based on USDA guidelines, it also signaled diet-related functional impairment and distress for the protagonist, but was nonetheless largely perceived as a healthy diet, as a “clean” diet, and more than moderately likely to be adopted by participants. Second, participant ratings of the average perceived healthiness, “cleanliness,” and willingness to adopt the “clean” diets depicted in the experimental vignettes were all significantly and moderately correlated with their scores on an ED screener, a measure of orthorexia nervosa, and a measure of preoccupation with body weight and fat. Thus, maintaining favorable attitudes toward “clean” diets to the detriment of social, school, and emotional functioning may reflect underlying eating psychopathology and indicate some overlap between “clean eating” and disordered eating. Indeed, the most highly rated reasons for the protagonist’s adoption of the “new” (balanced) diet were “to be healthier,” “for weight loss,” and “to feel in control of their diet,” of which the latter two rationales are also frequently associated with ED psychopathology. Unexpectedly, associations between attitudes toward the “clean” vignettes and indicators of obsessive-compulsive behaviors (OCI-R), weight bias internalization (WBIS-M), and body satisfaction (MBSRQ-AS-BASS) were small and largely non-significant, suggesting that there may also be some important differences between individuals who endorse “clean eating” versus those with EDs.

Given the initial, exploratory nature of the current investigation, there are several study limitations that offer useful opportunities for further research. First, in the absence of scholarly information regarding the “clean eating” cultural phenomenon, we relied heavily on popular media accounts and clinical anecdotes and included lifestyle and dietary elements (i.e., scenarios where the protagonist embraced all elements of “clean eating”) to create our experimental vignettes. It is therefore possible that we failed to assess other diets that may be categorized under the broad umbrella of “clean eating,” or that there may be instances where people participate in “clean” diets in a more flexible manner without the associated functional impairment and emotional distress. Future research using different methodologies such as “big data” strategies like social network analysis [49] or content analysis of hash-tagged images on Instagram [50] may further clarify the overall nature, communication strategies, and impact of the “clean eating” phenomenon. Alternately, further research assessing the relationship between “clean eating” and orthorexia nervosa, such as the applicability of proposed diagnostic criteria for ON [16, 19], focusing on clearer measurement given the lack of clarity regarding the measurement of ON [51–53], or examining indicators of psychopathology among self-identified “clean dieters” would help further our understanding of the possible benefits, harms, and overlap across the spectrum of healthy to disordered eating. Moreover, these findings have limited ecological validity and it is unclear how participants would perceive “clean” dieters in their everyday social interactions, or perceive those who subscribe to some (but perhaps not all) elements of the “clean eating” phenomenon. Finally, the present study focused solely on U.S.-based undergraduate students’ understanding of “clean eating”; although this was intentional, given the heightened risk for EDs on college campuses, it is possible that others have different reactions to the term and these require further exploration.

There has been a recent call to shift focus for ED prevention from targeted, individual-level programming to broader, public health level opportunities for change. For instance, Austin and colleagues [54] noted that “individual-level behavior change is ultimately essential for eating disorder prevention, but the neglect of macro-environmental targets undermines the potential for large-scale population impact” (p. 9) [54]. Further research on the merits and risks conferred by “clean eating” would clarify the opportunities to regulate the marketing and sale of “clean” dietary products (such as cleanses and supplements) to reduce risk among vulnerable groups. Moreover, scholarly literature could facilitate informed decision-making on the part of consumers of so-called “clean” diets. For instance, one scholarly paper touting the health benefits of the Alkaline diet [45], upon closer examination, was found to focus on pseudoscientific concepts such as “earthing” and was published in a medical journal for which the editor-in-chief had his medical license revoked by the State of Texas, by a publisher flagged as “potentially predatory” by Beall’s List of Predatory Journals and Publishers [55]. Scientific research is therefore needed to counteract the potentially harmful effects of the proliferation of pseudoscientific advocacy of dietary trends.

In sum, our preliminary findings suggest that U.S.-based young adults interpret “clean eating” in heterogenous but largely favorable ways, conferring various positive impacts and few harmful consequences to this dietary strategy. These results highlight the need to train consumers to better distinguish between trustworthy versus fraudulent sources of nutrition information and health behaviors (e.g., [56]). Although perceptions of specific diets commonly marketed as part of “clean eating” are variable, with some viewed as “cleaner” and healthier (such as the nutritionally balanced diet, or the vegan diet), and others rated much lower on these indicators (such as the meal substitution diet), the desire for weight loss and control over one’s diet are amongst the most highly rated reasons for adoption across “clean” diets. Moreover, it is concerning that our respondents exhibited positive attitudes toward an extreme “clean eating” regimen that caused distress and disruption across several indicators of functioning. It is also concerning that favorable perceptions of rigidly-maintained “clean” diets were linked with indicators of eating disorders and related symptomatology. Taken together, these findings highlight the need for ongoing examination of the “clean eating” phenomenon to clarify the potential benefits and risks posed by this dietary strategy and thereby inform ED prevention efforts on a broad level.

## Appendix 1

**Sample vignette**

Alex has been following **an alkaline diet** for the past three months after hearing about the merits of the alkaline diet from friends. An alkaline diet consists of avoiding all acid-forming foods such as meat, fish, and most legumes; people who are on an alkaline diet consume food such as vegetables, fruits, and certain nuts and seeds to help balance the pH levels of the blood. Alex does not have any intolerances or allergies that require following this type of diet. Instead, Alex believes that following an alkaline diet is a way to become more pure and healthy.

Alex has been having a hard time maintaining an alkaline diet: it has required more time, effort, and planning to find alkaline foods than anticipated, and this is starting to get in the way of Alex’s schoolwork. Moreover, when Alex breaks the rules for the alkaline diet, feelings of shame, guilt, and anxiety inevitably follow. The diet is even starting to negatively impact Alex’s social life. Since Alex started the diet, others have started to find mealtimes with Alex to be a little annoying. They do not appreciate Alex’s sense of superiority, which seems to stem from the alkaline diet. They are also concerned about the impact of the diet on Alex’s health and have asked Alex (on several occasions) to quit the diet. Despite these challenges, Alex feels good about the alkaline diet and does not understand why everyone does not follow an alkaline diet.

*Please answer the following question to ensure that you have understood the above scenario:*

The alkaline diet:

- Is the same thing as a vegan diet
- Consists of avoiding meat and dairy
- Is basically a vegetarian diet plus fish
- Consists of eating food that balances the pH level of the blood

## Abbreviations

ALK: Alkaline diet
BASS: Body Areas Satisfaction Scale
BMI: Body mass index (kg/m^2^)
CSPI: Center for science in the public interest
EDE-QS: Eating disorder examination - questionnaire short
EDs: Eating disorders
EHQ: Eating habits questionnaire
GF: Gluten-free diet
IRB: Institutional Review Board
MBSRQ-AS: Multidimensional body-self relations questionnaire - appearance scales
MS: Meal substitution diet
ND: “New” (balanced) diet
OCD: Obsessive-compulsive disorder
OCI-R: Obsessive-compulsive inventory - revised
ON: Orthorexia Nervosa
OP: Overweight preoccupation scale
USDA: United States Department of Agriculture
VG: Vegan diet
WBIS-M: Weight Bias internalization scale - modified

## Footnotes

1 One other empirical study on consumer food purchase behaviors [57] used the language of “eating clean” in the title, however, the article focused solely on organic food and there was no further mention of “clean eating” in the rest of the article.
